# Special Issue: Flavoenzymes

**DOI:** 10.3390/molecules23081957

**Published:** 2018-08-06

**Authors:** Willem J.H. van Berkel

**Affiliations:** Laboratory of Biochemistry, Wageningen University & Research, Stippeneng 4, 6708 WE Wageningen, The Netherlands; willem.vanberkel@wur.nl; Tel.: +31-6-12077313; Fax: +31-317-484801

The 19th International Symposium on Flavins and Flavoproteins was held from 2–6 July 2017 in Groningen, The Netherlands. The symposium, with 165 participants, highlighted recent developments in the field of flavin-dependent proteins and processes. In addition to various lectures by invited experts, the program included oral presentations selected based on submitted abstracts, young investigator talks, poster pitch talks, and posters. The vibrancy of the field was highlighted in sessions on: flavin-based chemistry; newly discovered flavoproteins; flavoenzyme engineering; flavoenzyme mechanisms and structures; flavoproteins and light; flavoenzymes and health, flavoenzymes and industrial applications.

The symposium took place in a special setting. The introductory lecture by Sandro Ghisla (University of Konstanz) about the origin of flavins, the session about flavin-based chemistry, the dinner lecture by Russ Hille (University of California) about flavins, flavoproteins, and flavinologists, and the closing PubQuiz about flavin facts and fiction were organized in the historic parish church Der Aa-kerk. All other sessions took place in Groninger Forum, a modern cinema, where an impressive iron wire model of *para*-hydroxybenzoate hydroxylase, one of the first flavoenzymes with a known three-dimensional structure, was on display during the entire conference. Many thanks to the local organizing team for creating such a splendid atmosphere.

Flavoenzymes are widespread in nature and catalyze a huge variety of redox reactions [1]. This versatility is well reflected in the Special Issue of *Molecules* dedicated to this conference. The compilation report features 13 research papers, dealing with flavoprotein monooxygenases, reductases, dehydrogenases, synthases, and oxidases. In particular, the last group of enzymes is well represented, which might be indicative of the increasing interest in flavin-mediated biocatalysis.

Albachelin is a hydroxamate-containing siderophore with an ornithine-based backbone that coordinates the iron. Bufkin and Sobradro report on the purification and initial characterization of albachelin monooxygenase, a novel *N*-hydroxylase from *Amycolatopsis alba* [2]. They show that the flavin adenine dinucleotide (FAD)-dependent enzyme hydroxylates ornithine and that lysine stimulates the oxidase activity of the enzyme. 

Castellano and Molinier-Frenkel give an overview of L-amino acid oxidase functions from bacteria to mammals with a focus on the immunoregulatory properties of phenylalanine oxidase IL4I1 [3]. They report that IL4I1 inhibits T-cell proliferation and cytokine production, and is expressed in the tumor-associated macrophages of most human cancers. The authors suggest that such expression, associated with its capacity to facilitate tumor growth by inhibiting the anti-tumor T-cell response, makes IL4I1 a new potential druggable target in the field of immunomodulation in cancer.

The FAD-containing 5-(hydroxymethyl)furfural oxidase from *Methylovorus* sp. MP688 has the remarkable capability of oxidizing [5-(hydroxymethyl)furan-2-yl]methanol to the bio-based platform chemical furan-2,5-dicarboxylic acid via four sequential oxidation steps. Pickl et al. describe the rational engineering of 5-(hydroxymethyl)furfural oxidase for the improved direct oxidation of alcohols to carboxylic acids [4]. After exchanging two active-site residues for hydrogen-bond donating and accepting amino acids, enhanced over-oxidation was demonstrated.

FAD-dependent L-amino acid oxidases catalyze the oxidative deamination of L-amino acids, forming the corresponding keto acids. Hahn et al. found that the activity of L-amino acid oxidase from *Rhizoctonia solani* is strongly stimulated in the presence of low concentrations of the detergent sodium dodecyl sulfate [5]. Spectroscopic experiments led the authors to suggest that the sodium dodecyl sulfate-activated enzyme has a more open conformation facilitating access to the active site.

Proline utilization A (PutA) proteins are bifunctional enzymes that catalyze the oxidation of l-proline to l-glutamate. Korasick et al. describe the structural basis for the substrate inhibition of PutA by proline [6]. From the X-ray diffraction data and kinetic studies, they conclude that the substrate inhibition of the PutA coupled reaction is due to proline binding in the L-glutamate-semialdehyde dehydrogenase active site.

Ferredoxin-NADP(H) reductases (FNRs) deliver NADPH or low potential one-electron donors to redox-based metabolism in plastids and bacteria. Martinez-Júlvez et al. present data about the identification of inhibitors targeting FNR from the phytopathogenic bacterium *Xanthomonas citri* subspecies *citri* (*Xcc*) [7]. Using high-throughput screening and inhibition kinetics, they find several FNR inhibitors that can become interesting tools to discover *Xcc* antimicrobials.

Acyl-CoA dehydrogenases are FAD-dependent enzymes that catalyze the oxidation of α,β-carbon bonds in acyl-CoA thioesters. Burgener et al. report on the molecular basis for converting (2*S*)-methylsuccinyl-CoA dehydrogenase into an oxidase [8]. Using stopped-flow absorption spectroscopy- and liquid chromatography-mass spectrometry-based assays, they show that the increased oxidase function of the engineered enzyme coincides with decreased dehydrogenase activity. The authors suggest that solvent accessibility, channeling, and gate-keeping all contribute to the observed effects and that their findings provide guidelines for rational engineering efforts of acyl-CoA dehydrogenases and oxidases.

The vanillyl alcohol oxidase/*para*-cresol methylhydroxylase (VAO)/PCMH) flavoprotein family consists mostly of oxidoreductases harboring a covalently linked flavin cofactor [9]. Ferrari et al. describe the initial characterization of two VAO-type flavoprotein oxidases from the thermophilic mold *Myceliophthora thermophila* C1 [10]. MtVAO615 was found to contain a bi-covalently bound FAD, while MtVAO713 contains a mono-covalent histidyl-bound FAD. Both proteins, when reduced, rapidly react with molecular oxygen, but their crystal structures show atypical active site architectures, hampering the identification of actual substrates. During the screening of a library of potential substrates, low oxidase activity towards ricinoleic acid was discovered for MtVAO713.

FMN:ATP adenylyl transferase, commonly known as FAD synthase (FADS) is the last enzyme involved in the pathway of FAD biosynthesis. Leone et al. report the characterization of a novel isoform of human FADS, consisting of the sole C-terminal phosphoadenosine-phosphosulfate reductase domain [11]. This isoform has been previously detected in riboflavin-responsive and non-responsive multiple acyl-CoA dehydrogenase deficiency patients. The monomeric protein catalyzes FAD synthesis as well as FAD pyrophosphorolysis in a strictly Mg^2+^-dependent manner, but lacks the ability to hydrolyze FAD. The relevance of this FADS isoform to human physio-pathology is discussed.

Vanillyl alcohol oxidase (VAO) and eugenol oxidase (EUGO) are covalent FAD enzymes that catalyze the oxidation of a wide range of *para*-substituted phenols [9]. Ewing et al. present an efficient screening assay for the substrate specificity of *para*-phenol oxidases based on the detection of hydrogen peroxide using the ferric-xylenol orange complex method [12]. The screening of a small library of VAO and EUGO active-site variants with 24 potential substrates led to the identification of a novel substrate (4-cyclohexylphenol) and increased activities with vanillyl alcohol, chavicol, and 4-cyclopentylphenol.

Proline dehydrogenase (ProDH) is a ubiquitous flavoenzyme that catalyzes the oxidation of proline to Δ^1^-pyrroline-5-carboxylate, the initial step of proline catabolism [6]. ProDH from *Thermus thermophilus* (TtProDH) contains, in addition to its flavin-binding TIM-barrel domain, an N-terminal arm, consisting of helices A, B, and C. Huijbers et al. report that the truncated TtProDH variants ΔA and ΔAB are highly active tetramers [13]. The removal of the entire N-terminal arm (ΔABC) results in barely active dimers. The characterization of V32D, Y35F, and V36D variants of ΔAB established that a hydrophobic patch between helix C and helix 8 is critical for TtProDH catalysis and tetramer stabilization.

Flavin-dependent nitroreductases (NRs) hold promise for converting nitroaromatics to pharmaceutically relevant aromatic amines. Miller et al. report that four NR subgroups provide contrasting substrate binding cavities with distinct constraints on substrate position relative to the flavin [14]. Based on the unique architecture of the NR dimer, they propose that the functional diversity included in the NR superfamily stems from the chemical versatility of the flavin cofactor in conjunction with a structure that permits tremendous active site variability. These complementary properties make NRs exceptionally promising enzymes for the development of biocatalysis in prodrug activation and the conversion of nitroaromatics to valuable aromatic amines.

Styrene monooxygenases (SMOs) are two-component flavoproteins that catalyze regio- and enantioselective epoxidation and sulfoxidation reactions. Tischler et al. describe the first representative of an E2-type two-component styrene monooxygenase of proteobacteria [15]. It comprises a single epoxidase protein (VpStyA1) and a two-domain protein (VpStyA2B) harboring an epoxidase (A2) and a FAD-reductase (B) domain. The authors show that VpStyA1/VpStyA2B of *Variovorax paradoxus* strain EPS is an aryl alkyl sulfoxidase rather than a styrene epoxidizing monooxygenase and that VpStyA1 is the most active component of the system, producing dominantly (*S*)-enantiomeric sulfoxides.

The contributions to this Special Issue provide a good picture of the chemical virtuosity of flavins and the biochemical diversity of flavoenzymes. It is clear that more exciting flavin news may be expected in the near future. With that in mind, we look forward to the 20^th^ International Symposium on Flavins and Flavoproteins in July 2020 in Graz. More details about this conference will be announced at www.flavoproteins.com.

## References

[1] Van Berkel W.J.H., Begley T. (2008). Chemistry of flavoenzymes. Wiley Encyclopedia of Chemical Biology.

[2] Bufkin K., Sobrado P. (2017). Characterization of the ornithine hydroxylation step in albachelin biosynthesis. Molecules.

[3] Castellano F., Molinier-Frenkel V. (2017). An overview of l-amino acid oxidase functions from bacteria to mammals: Focus on the immunoregulatory phenylalanine oxidase IL4I1. Molecules.

[4] Pickl M., Winkler C.K., Glueck S.M., Fraaije M.W., Faber K. (2017). Rational engineering of a flavoprotein oxidase for improved direct oxidation of alcohols to carboxylic acids. Molecules.

[5] Hahn K., Hertle Y., Bloess S., Kottke T., Hellweg T., Fischer von Mollard G. (2017). Activation of recombinantly expressed l-amino acid oxidase from *Rhizoctonia solani* by sodium dodecyl sulfate. Molecules.

[6] Korasick D.A., Pemberton T.A., Arentson B.W., Becker D.F., Tanner J.J. (2018). Structural basis for the substrate inhibition of proline utilization A by proline. Molecules.

[7] Martínez-Júlvez M., Goñi G., Pérez-Amigot D., Laplaza R., Ionescu I.A., Petrocelli S., Tondo M.L., Sancho J., Orellano E.G., Medina M. (2018). Identification of inhibitors targeting ferredoxin-NADP^+^ reductase from the *Xanthomonas citri* subsp. *citri* phytopathogenic bacteria. Molecules.

[8] Burgener S., Schwander T., Romero E., Fraaije M.W., Erb T.J. (2018). Molecular basis for converting (2*S*)-methylsuccinyl-CoA dehydrogenase into an oxidase. Molecules.

[9] Ewing T.A., Fraaije M.W., Mattevi A., van Berkel W.J.H. (2017). The VAO/PCMH flavoprotein family. Arch. Biochem. Biophys..

[10] Ferrari A.R., Rozeboom H.J., Vugts A.S.C., Koetsier M.J., Floor R., Fraaije M.W. (2018). Characterization of two VAO-type flavoprotein oxidases from *Myceliophthora thermophila*. Molecules.

[11] Leone P., Galluccio M., Barbiroli A., Eberini I., Tolomeo M., Vrenna F., Gianazza E., Iametti S., Bonomi F., Indiveri C. (2018). Bacterial production, characterization and protein modeling of a novel monofuctional isoform of FAD synthase in humans: An emergency protein?. Molecules.

[12] Ewing T.A., van Noord A., Paul C.E., van Berkel W.J.H. (2018). A xylenol orange-based screening assay for the substrate specificity of flavin-dependent *para*-phenol oxidases. Molecules.

[13] Huijbers M.M.E., van Alen I., Wu J.W., Barendregt A., Heck A.J.R., van Berkel W.J.H. (2018). Functional impact of the *N*-terminal arm of proline dehydrogenase from *Thermus thermophilus*. Molecules.

[14] Miller A.F., Park J.T., Ferguson K.L., Pitsawong W., Bommarius A.S. (2018). Informing efforts to develop nitroreductase for amine production. Molecules.

[15] Tischler D., Schwabe R., Siegel L., Joffroy K., Kaschabek S.R., Scholtissek A., Heine T. (2018). VpStyA1/VpStyA2B of *Variovorax paradoxus* EPS: An aryl alkyl sulfoxidase rather than a styrene epoxidizing monooxygenase. Molecules.

